# Prognostic significance of the get with the guidelines-heart failure (GWTG-HF) risk score in patients undergoing trans-catheter tricuspid valve repair (TTVR)

**DOI:** 10.1007/s00380-021-01874-3

**Published:** 2021-05-22

**Authors:** Refik Kavsur, Hannah Emmi Hupp-Herschel, Atsushi Sugiura, Tetsu Tanaka, Can Öztürk, Marcel Weber, Georg Nickenig, Vedat Tiyerili, Marc Ulrich Becher

**Affiliations:** Heart Center Bonn, Department of Medicine II, University Hospital Bonn, Venusberg-Campus 1, 53127 Bonn, Germany

**Keywords:** Tricuspid regurgitation, Heart failure, Tricuspid valve

## Abstract

The Get-With-The-Guidelines-Heart-Failure (GWTG-HF) score is a risk assessment tool to predict mortality in patients with heart-failure (HF). We aimed to evaluate the GWTG-HF score for risk stratification in HF patients with tricuspid regurgitation undergoing trans-catheter tricuspid valve repair (TTVR). In total, 181 patients who underwent TTVR via edge-to-edge repair (86%) or annuloplasty (14%) were enrolled. Patients were categorized into a low- (≤ 43 points), intermediate- (44–53 points) and high-risk score groups (≥ 54 points). TTVR led to an improvement of TR (*p* < 0.0001) and NYHA (*p* < 0.0001). Kaplan–Meier analysis and log-rank test revealed that higher GWTG-HF scores were associated with reduced rates of event-free survival regarding mortality (96% vs 89% vs 73%, respectively, *p* = 0.001) and hospitalization for heart failure (HHF) (89% vs 86% vs 74%, respectively, *p* = 0.026). After adjusting for important variables like renal function, left ventricular ejection fraction and mitral regurgitation, the GWTG-HF score remained an independent predictor of the composite endpoint of HHF or mortality (hazard ratio 1.04 per 1-point increase, *p* = 0.029). Other remaining predictors were renal function and mitral regurgitation. The GWTG-HF score used as a risk stratification tool of mortality and HHF maintains its prognostic value in a HF population with severe TR undergoing TTVR.

**Supplementary Information:**

The online version contains supplementary material available at 10.1007/s00380-021-01874-3.

## Introduction

Trans-catheter repair of severe tricuspid regurgitation (TR) is an emerging field, representing a minimal-invasive alternative to tricuspid valve surgery, which is associated with poor morbidity and mortality rates [1]. For trans-catheter tricpuspid valve repair (TTVR), randomized clinical trials are in need, however, first trials and observational studies showed promising results [2–5]. Optimized patient selection may be crucial for TTVR and risk stratification tools are missing.

The Get-with-The-Guidelines-Heart Failure (GWTG-HF) risk score was initially introduced for prediction of in-hospital mortality in acute heart failure (HF) patients [6]. In a following validation study, an improvement of its predictive ability was shown by the inclusion of B-type natriuretic peptide level [7].

Beyond that, the GWTG-HF score demonstrated its use for risk stratification in patients with chronic HF and regarding post-discharge events [8, 9]. Moreover, a recent study revealed a high correlation of this score with prognosis in patients undergoing trans-catheter mitral valve repair [10]. Due to their close interaction, TR is common in HF and portends poor prognosis [11]. Therefore, the GWTG-HF score might be particularly useful in predicting the risk of poor outcome in HF patients undergoing TTVR for severe TR.

The aim of the present study was to evaluate the prognostic significance of the GWTG-HF score in symptomatic HF patients with severe TR undergoing TTVR regarding all-cause mortality and hospitalization for HF (HHF). In addition, we assessed the predictive power after the inclusion of serum levels of n-terminal pro-B-type natriuretic peptide (NT-proBNP).

## Methods

### Study population

One-hundred-and-eighty-one patients undergoing TTVR between June 2015 and October 2020 were included. All patients went through an evaluation process for TTVR including diagnostics, such as transthoracic/transoesophageal echocardiography and left heart catheterization. After completion, all patients were discussed in the interdisciplinary heart team and considered to be at a high surgical risk. Participants of this present study agreed to be included in our registry which was approved by the Ethical Committee of the University Hospital Bonn, and gave written informed consent.

### GWTG-HF score

Calculation of the GWTG-HF score was performed as previously reported [6, 12], using the following 7 predictor variables, which were available for all patients prior to TTVR: age, race, systolic blood pressure, heart rate, serum urea nitrogen, sodium and chronic obstructive pulmonary disease. The score ranged between 0 and 100 points and was assessed for each patient.

### Procedures

Two different approaches were used for TTVR: Trans-catheter edge-to-edge repair via MitraClip (Abbott Vascular, Santa Clara, CA, USA) or trans-catheter annuloplasty via Cardioband (Edwards Life-sciences, Irvine, CA, USA). Procedural decisions were left to the discretion of the operators. All interventions were performed under general anesthesia. During the intervention, transoesophageal echocardiography and fluoroscopy were used for guidance. Echocardiograms were analyzed in concordance with current international recommendations and involved TR grading in five stages (grade 1–5) [13, 14]. Primary procedural success was defined as interventional reduction of at least one grade in TR severity. Regarding MR, there were 48/181 (27%) patients with moderate MR, 19/181 (11%) patients with moderate-to-severe MR, and 3/181 (2%) patients with severe MR. All three patients with concomitant severe MR got combined repair of TR and MR.

### Follow-up and outcome

After TTVR, patients were followed up via regular visits which were scheduled at 30 days and every 3–6 months. These visits included clinical examination, echocardiography and assessment of New York Heart Association (NYHA) class. In cases of missing follow-ups, the patient, the patient’s general practitioner or cardiologist was contacted via telephone. Primary endpoints included all-cause mortality, hospitalization for HF and the combined endpoint composite of both, within a year follow-up after TTVR.

### Statistical analysis

Kolmogorov–Smirnov test was performed to test normality. Comparing normally distributed, continuous variables, Student’s *t* test, ANOVA (unpaired) or paired *t* test were used. For continuous variables which were not normally distributed, Mann–Whitney *U* test, Kruskal–Wallis (unpaired) or Wilcoxon test (paired) was performed. Variable association with outcome was assessed using Cox proportional hazard analysis. For multivariable Cox analysis, variables were involved which showed a significant predictive value in the uni-variable Cox analysis, excluding parameters which were included in the GWTG-HF score. Event-free survival rates were plotted using Kaplan–Meier curves and differences were analyzed, using the log-rank test. Receiver operating characteristics curve (ROC) analyses were performed to determine the predictive power of the GWTG-HF score and evaluate a cut-off by comparing changes in area under curve (AUC). To evaluate the impact of NT-proBNP on risk stratification, logistic regression analysis was used, subsequently probability values were included into ROC analysis. *p *value of < 0.05 was considered to be statistically significant. Statistical analysis was conducted with SPSS Statistics software version 24.0.0.0 (IBM, Armonk, NY, USA).

## Results

### Patient cohort

A total of 181 patients who underwent TTVR were included into final analysis. Patient cohort’s mean age was 77 ± 7 and 59% were of female gender. LVEF was 57% (51–62%) and NT-proBNP was 2033 ng/l (1016–3804 ng/l). Mean logistic EuroSCORE of 15.9% (8.5–27.5%) revealed a high surgical risk profile of the patient cohort. Baseline characteristics are summarized according to GWTG-HF score in Table 1.

**Table 1:** Baseline characteristics

	GWTG-HF score			*p* Value
	Low *n*=56	Intermediate *n*=63	High *n*=62	
Clinical characteristics				
Age, years	77 (68–80)	79 (76–83)	80 (75–83)	**0.002**
Systolic BP, mmHg	140 (130–153)	120 (110–130)	115 (110–130)	**<0.0001**
Heart rate, per min	65 (56–75)	71 (64–80)	72 (60–81)	**0.040**
Female gender	68% (38)	59% (37)	52% (32)	0.200
BMI, kg/m²	24.8 (22–28.4)	25.4 (22.5–28.7)	25.3 (21.8–27.0)	0.794
Log EuroSCORE, %	13 (6–20)	16 (8–27)	19 (12–34)	**0.001**
Diabetes	21% (12)	27% (17)	32% (20)	0.417
Prior stroke	9% (5)	14% (9)	16% (10)	0.493
COPD	9% (5)	30% (19)	23% (14)	**0.017**
History of smoking	21% (12)	27% (17)	29% (18)	0.626
Coronary artery disease	52% (29)	57% (36)	605 (37)	0.681
Prior CABG	20% (11)	25% (16)	27% (17)	0.598
Prior valvular surgery	43% (24)	35% (22)	36% (22)	0.615
Atrial fibrillation	89% (50)	91% (57)	94% (58)	0.628
Lead	29% (16)	29% (18)	40% (25)	0.278
NYHA class >II	89% (50)	87% (55)	86% (53)	0.826
Peripheral artery disease	33% (18)	40% (25)	60% (36)	**0.009**
Carotid stenosis	7% (4)	16% (10)	17% (10)	0.241
Echocardiographic data				
TR ≥ IV	41% (23)	44% (28)	48% (30)	0.726
Functional TR etiology	100% (56)	97% (61)	97% (60)	0.400
Annuloplasty	21% (12)	11% (7)	11% (7)	0.201
Procedural success	85% (46)	77% (48)	88% (53)	0.247
LVEF, %	57 (49–61)	57 (53–64)	57 (51–62)	0.754
MR ≥ II	36% (20)	44% (28)	36% (22)	0.507
TAPSE, mm	16 (14–20)	18 (15–22)	17 (13–20)	0.158
Systolic PAP, mmHg	35 (30–48)	32 (25–40)	34 (26–42)	0.383
Laboratory assessment				
Sodium mmol/l	140 (138–141)	139 (137–142)	138 (135–140)	**0.004**
NT‐proBNP, pg/ml	1313 (877–2142)	2040 (1069–2998)	3300 (1915–5331)	**<0.0001**
GFR, ml/min	60 (47–70)	48 (38–68)	32 (26–46)	**<0.0001**
Blood urea nitrogen, mg/dl	39 (32–49)	55 (41–73)	110 (86–144)	**<0.0001**
Hemoglobin, g/dl	12 (11–13)	12 (10–13)	11 (10–13)	0.058

Differences with a *p* value < 0.05 were considered as statistically significant (bold). Values are % (*n*), or median (interquartile range)

*BMI* body mass index, *BP* blood pressure, *CABG* coronary artery bypass grafting, *COPD* chronic obstructive pulmonary disease, *GFR* estimated glomerular filtration rate, *LVEF* left ventricular ejection fraction, *MR* mitral regurgitation, *NYHA* New York Heart Association class, *PAP* pulmonary artery pressure, *TAPSE* tricuspid annular plane systolic excursion, *TR* tricuspid regurgitation

### Procedural data and feasibility

TTVR included catheter-based annuloplasty of the tricuspid valve in 26 (14%) patients and edge-to-edge repair in 154 (86%) patients: edge-to-edge repair was performed in 90% for isolated TR and 10% for TR and MR. Primary procedural success (reduction of one grade in TR severity) was accomplished for 153 (85%) patients. In total, 202 clips were implanted with a mean of 1.6 ± 1.0 per edge-to-edge repair.

At baseline, eleven patients (6%) had a TR grade which was moderate to severe, while 170 patients (94%) had at least a severe TR. Post-procedural frequencies of TR ≥ III reduced to 21% at one-month follow-up and remained at 23% at 6-month follow-up. Median TR grade decreased from 3 (3–4) at baseline to 2 (1–3) at one month (*p* < 0.0001). Moreover, median NYHA of 3 (3–3) improved to a median NYHA of 2 (1–2) at one-month follow-up (*p* < 0.0001).

### Comparison of the GWTG-HF score parameters

**Fig. 1:**
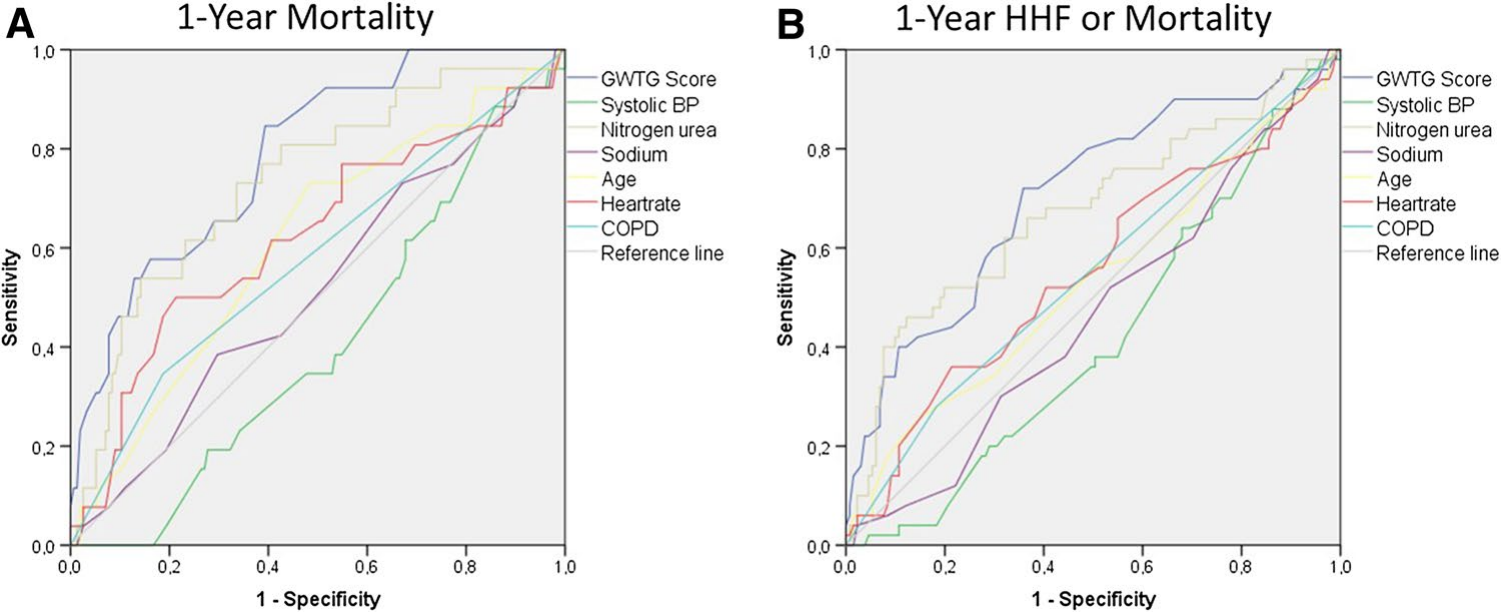
Receiver operating characteristic curve. The Get-With-The-Guidelines-Heart Failure Score (GWTG-HF) revealed a higher predictive value with an area under curve (AUC) of 0.788 [(0.701–0.876), *p* < 0.0001] regarding 1-year mortality (**A**), and 0.706 [(0.618–0.793), *p* < 0.0001] regarding composite endpoint of 1-year heart failure hospitalization (HHF) or mortality (**B**), comparing with the scores individual parameters.

Mean GWTG-HF score was 49 ± 9. Patients were categorized into a low- (*n* = 56 with ≤ 43 points), intermediate- (*n* = 63 with 44–53 points) and high-risk score groups (*n* = 62 with ≥ 54 points) according to GWTG-HF score tertiles. At baseline, median log EuroSCORE increased with increasing GWTG-HF score groups [13% (6–20%), 16% (8–27%) and 19% (12–34%), respectively, *p* = 0.001], and peripheral artery disease was more prevalent in the higher score groups (33%, 40% and 60%, respectively, *p* = 0.009). Moreover, estimated serum glomerular filtration rate (*p* < 0.0001) decreased and NT-proBNP (< 0.0001) increased with increasing GWTG-HF score group. Comparing six (excluding race) parameters of the GWTG-HF score, all parameters showed expected differences comparing the three groups (Table 1). AUC values from ROC analysis of GWTG-HF score and its individual parameters are shown in Supplementary Table 1. In this analysis regarding 1-year mortality, and the combined endpoint of HHF or mortality, the GWTG-HF score revealed best AUC by a clear margin [0.788 (0.701–0.876), *p* < 0.0001 & 0.706 (0.618–0.793), *p* < 0.0001, respectively] (Fig. 1).

### Impact of GWTG-HF score on outcome

**Fig. 2:**
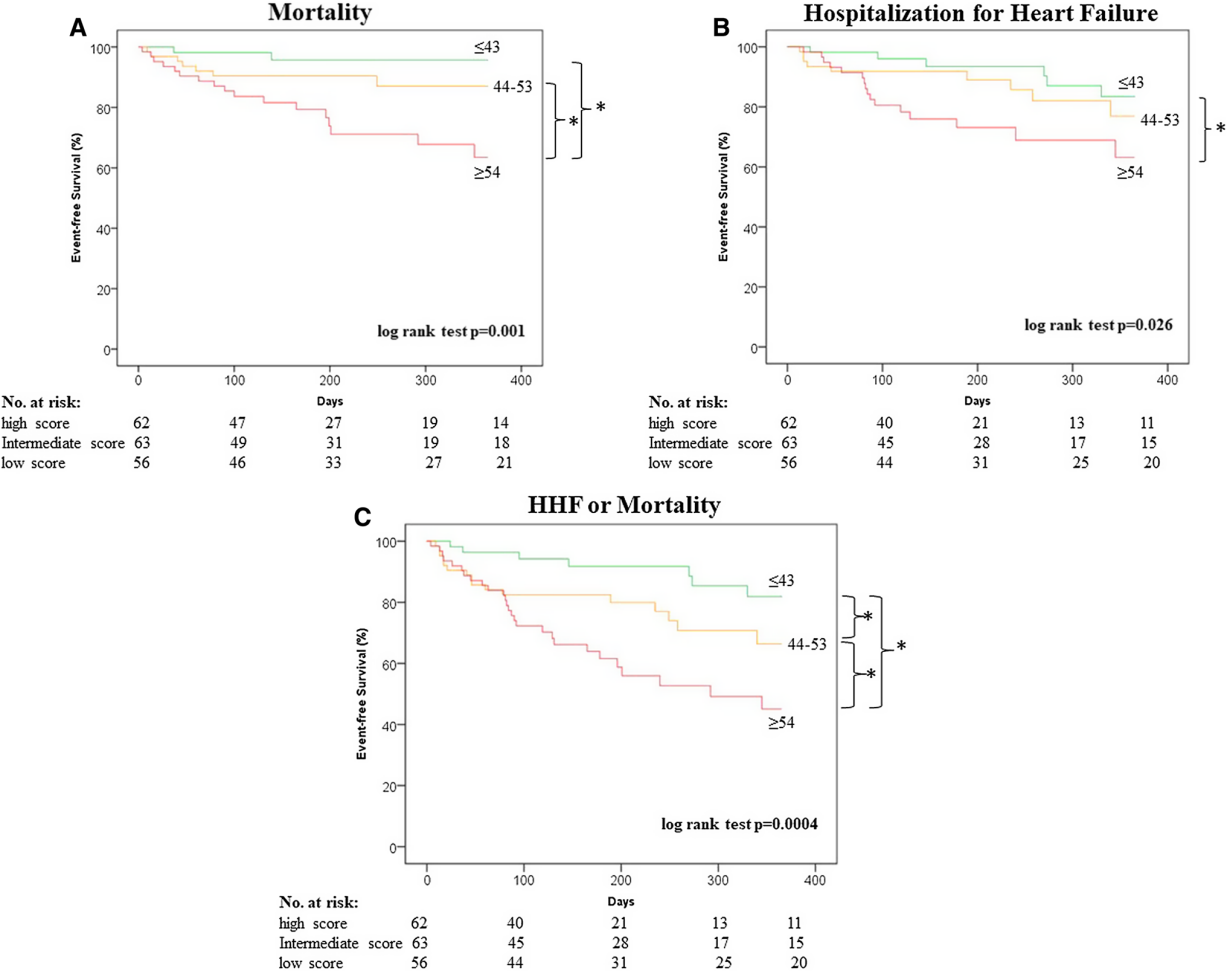
Survival and Hospitalization for Heart Failure According to GWTG-HF Score. Patients undergoing trans-catheter tricuspid valve repair showed increasing rates of **A** all-cause mortality, **B** hospitalization for heart failure (HHF), and **C** the composite endpoint of both with increasing Get With The Guidelines-Heart Failure (GWTG-HF) score (green = low, orange = intermediate, red = high score). *p < 0.05

During 1-year follow-up after TTVR, 26 (14%) patients died and 31 (17%) were readmitted for HF. Kaplan–Meier analysis and log-rank test showed lower event-free survival with increasing GWTG-HF score group, from 1-year all-cause mortality (96% vs 89% vs 73%, respectively, log-rank test *p* = 0.001) (Fig. 2A), HHF (89% vs 86% vs 74%, respectively, log-rank test *p* = 0.026) (Fig. 2B) and the composite endpoint of HFF and mortality (88% vs 75% vs 57%, respectively, log-rank test, *p* = 0.0004) (Fig. 2C). Cox regression analysis of the GWTG-HF score and other relevant parameters are presented in Supplementary Table 2. This analysis revealed that one increase in the GWTG-HF score was associated with a 1.11-fold (95% CI, 1.003–1.10, *p* < 0.0001) higher rate of mortality and a 1.07-fold (95% CI, 1.04–1.11, *p* < 0.0001) increased rate of the combined endpoint of HHF or mortality (per 1-point increase). After adjusting for clinically important variables including LVEF, mitral regurgitation and glomerular filtration rate, the GWTG-HF score remained an independent predictor of the combined endpoint of HHF and mortality (hazard ratio 1.04, 95% CI, 1.004–1.08, *p* = 0.029) (per 1-point increase). Other predictive markers were mitral regurgitation [hazard ratio 1.97 (95% CI, 1.14–3.41, *p* = 0.016)] and estimated glomerular filtration rate [hazard ratio 0.98 (95% CI, 0.95–1.0) per 1 ml/min increase, *p* = 0.043], while LVEF [hazard ratio 0.97 (95% CI, 0.95–1.0) per 1% increase, *p* = 0.053], tricuspid annular plane systolic excursion [hazard ratio 0.093 (95% CI, 0.86–1.01) per 1 mm increase, 0.089], and procedural success [hazard ratio 0.5 (95% CI, 0.24–1.06), *p *= 0.069] had values which marginally exceeded threshold of statistical significance (Table 2).

**Table 2:** Predictor of heart failure hospitalization/mortality

	HHF or mortality	
Predictor	Hazard ratio	*p* Value
GWTG-HF score (per 1-point increase)	1.04 (1.004–1.08)	0.029
GFR (per 1 ml/min increase)	0.98 (0.95–1.0)	0.043
Nt-pro-BNP (per 1000 pg/ml increase)	1.02 (0.98–1.06)	0.450
Mitral regurgitation (per 1 grade increase)	1.97 (1.14–3.41)	0.016
LVEF (per 1% increase)	0.97 (0.95–1.0)	0.053
Logistic EuroSCORE (per 1% increase)	1.01 (0.99–1.03)	0.582
TAPSE (per 1 mm increase)	0.93 (0.86–1.01)	0.089
Procedural success	0.5 (0.24–1.06)	0.069

*GFR* estimated glomerular filtration rate, *LVEF* left ventricular ejection fraction, *TAPSE* tricuspid annular plane systolic excursion

### Inclusion of NT-proBNP for risk stratification

We further assessed the impact of adding NT-proBNP to the GWTG-HF on the risk stratification ability of 1-year mortality and the combined endpoint of HHF or mortality. The inclusion of NT-proBNP to the GWTG-HF risk score resulted in an improvement of the predictive power for mortality [AUC 0.788 (0.701–0.876) vs AUC 0.799 (0.709–0.888)], and for the combined endpoint HHF or mortality [AUC 0.700 (0.607–0.794) vs AUC 0.708 (0.616–0.800)].

## Discussion

In the current study, we assessed the GWTG-HF score as a risk stratification tool in HF patients with severe TR undergoing TTVR regarding 1-year mortality and readmission for HF. We demonstrate (1) that TTVR including trans-catheter edge-to-edge repair and annuloplasty was feasible in the majority of patients and effective in substantial improvement of TR and NYHA class; (2) that patients displayed a worse event-free survival with increasing GWTG-HF score categorization, regarding mortality, HHF and the combined endpoint composite of both; (3) that each 1-point increase in the GWTG-HF score was independently associated with a 4% increase in the risk for the composite endpoint of mortality and HHF, after adjusting for parameters including LVEF, mitral regurgitation and renal function; and (4) that the high predictive power of the GWTG-HF score regarding outcome could be improved by the inclusion of NT-proBNP levels.

The GWTG-HF program by the American Heart Association is designed for improving the care of patients who are hospitalized for HF. One of the results of the program is the GWTG-HF risk score which represents a risk assessment tool for prediction of in-hospital mortality in patients with acute HF, irrespective of reduced or preserved left ventricular function [6]. The score consists of 7 variables which are commonly available in clinical practice and represent known risk factors of mortality or marker of advanced HF. In patients with HF, TR is common and displays a prognostic relevant comorbidity [15, 16]. There are recent data, demonstrating favorable outcomes in HF patients with symptomatic TR who underwent TTVR compared to medical treatment [3]. In consisting, the present study showed a substantial effect of TTVR on TR and NYHA improvement. We further demonstrate that in HF patients undergoing TTVR, the GWTG-HF score serves for risk stratification, identifying a high-risk cohort with impaired 1-year event-free survival. Patients with a low GWTG-HF score presented a favorable outcome regarding mortality and hospitalization for HF (Fig. 2). Moreover, multivariable analysis showed an independent association of the GWTG-HF score with mortality and the combined endpoint composite of death and HHF, after adjusting for clinically relevant variables which included LVEF, mitral regurgitation and renal function. The predictive ability was confirmed in ROC analysis revealing an AUC of 0.788 [(0.701–0.876), *p* < 0.0001 and 0.706 (0.618–0.793), *p* < 0.0001], which is considered in a comparable range of prior studies on this score [6, 7, 9]. Comparing this result with a recent study on this score in patients undergoing trans-catheter mitral valve repair [10], the present data revealed a higher AUC, underlining the utility of the GWTG-HF score for this population of TTVR patients. Multivariable analysis indicates that the score showed similar predictive power to more expectable clinical variables like renal function and mitral regurgitation, and moreover, showed superiority in comparison to parameters like NT-pro-BNP, LVEF, tricuspid annular plane systolic excursion and logistic EuroSCORE. This indicates that HF patients with severe TR may represent a distinct target of the GWTG-HF risk score. Of note is that the GWTG-HF score showed a higher AUC regarding both endpoints in comparison to its individual parameters, which indicates that the combination of its parameters leads to the relevant predictability.

The mechanisms, which lead to better outcome after TR reduction, are unknown. One important aspect involved, is that TTVR reduces backwards right ventricular failure thereby inhibiting fluid retention and congestion of the venous system, thus improving renal and liver function [3]. In this context, the cardio-renal syndrome is of particular interest, representing a form of renal failure which is associated with HF [17]. Regarding characteristics of our cohort at baseline (Table 1), comparing patients with a low, intermediate and high GWTF-HF scores, serum glomerular filtration rate and NT-proBNP were two parameters which were not included in the GWTG-HF score and still showed a highly significant difference between the three groups. Higher levels of blood urea nitrogen and inferior glomerular filtration rates indicate that patients with a high GWTG-HF score had a more advanced renal impairment. Therefore, a pronounced co-existing cardio-renal syndrome at baseline is likely for less beneficial outcomes after TTVR in these high GWTG-HF-patients.

NT-proBNP is a known prognostic marker of HF [18] and can be disturbed by concomitant renal dysfunction [19]. In the present study, the predictive power of the GWTG-HF score was improved by the inclusion of NT-proBNP. Because of the close interaction of HF prognosis, congestion and renal impairment, high GWTG-HF scores with or without inclusion of NT-proBNP reliably detected patient with high risk of cardiovascular events in our TTVR cohort. These high GWTG-HF score patients might have less benefit of TTVR most likely due to advanced stages of HF.

### Study limitations

In the present study which is one of the first investigating a risk assessment tool for TTVR in HF patients, several limitations must be noted. The study is single-centered and includes a limited sample size. However, considering the novelty of this emerging field, the present cohort represents a reasonable sample size compared to prior studies regarding TTVR. Furthermore, it is observational and non-randomized character, warrants cautious interpretation because of potential bias. Lastly, our cohort consisted of Caucasian patients, thus, relevance of race could not be assessed.

### Conclusion

The GWTG-HF score serves as a risk assessment tool in HF patients with concomitant severe TR undergoing TTVR to predict 1-year mortality and HHF. The score helps to identify patients with advanced risk of cardiovascular events due to HF who might need a more intensive monitoring and HF treatment after TTVR. Moreover, the inclusion of NT-proBNP led to an improvement of the GWTG-HF score’s predictive power, emphasizing its use in this particular HF patient’s population. Overall, in this present study, TTVR was feasible in the vast majority of patients and led to a substantial improvement of TR and NYHA classes.

## Supplementary Information

Below is the link to the electronic supplementary material.

Supplementary file1 (DOCX 17 kb)

## Abbreviations

AUC: Area under curve
COPD: Chronic obstructive pulmonary disease
GWTG-HF: Get-With-The-Guidelines-Heart Failure risk score
HF: Heart failure
HHF: Hospitalization for heart failure
HR: Hazard ratio
LVEF: Left ventricular ejection fraction
MR: Mitral regurgitation
NT-proBNP: N-terminal pro-B-type natriuretic peptide
NYHA: New York Heart Association
ROC: Receiver-Operating Characteristics curve
TR: Tricuspid regurgitation
