# Supplementary material for: Prognostic significance of the get with the guidelines-heart failure (GWTG-HF) risk score in patients undergoing trans-catheter tricuspid valve repair (TTVR)
Source: Heart Vessels. 2021 May 22;36(12):1903–10. doi: 10.1007/s00380-021-01874-3 (PMC8556194; doi:10.1007/s00380-021-01874-3)
Supplement: Supplementary file 1 — Supplementary file1 (DOCX 17 kb) [file 380_2021_1874_MOESM1_ESM.docx]

**Supplementary Material**

Supplementary Table 1. Predictive Value of Individual GWTG-HF Score Parameters

|  | Area Under Curve | | |  |
| --- | --- | --- | --- | --- |
| GWTG-HF Parameter | Mortality | p value | HHF or Mortality | p value |
| Age | 0.613 (0.497-0.729) | 0.066 | 0.533 (0.434-0.631) | 0.499 |
| Sodium | 0.517 (0.395-0.639) | 0.780 | 0.453 (0.371-0.557) | 0.453 |
| Heart rate | 0.623 (0.496-0.750) | 0.045 | 0.552 (0.454-0.650) | 0.283 |
| COPD | 0.580 (0.455-0.704) | 0.195 | 0.548 (0.452-0.644) | 0.315 |
| Systolic BP | 0.408 (0.302-0.515) | 0.136 | 0.419 (0.330-0.508) | 0.092 |
| Urea nitrogen | 0.736 (0.630-0.842) | 0.0001 | 0.674 (0.580-0.768) | 0.0003 |
| **GWTG-HF score** | 0.788 (0.701-0.876) | <0.0001 | 0.706 (0.618-0.793) | <0.0001 |

Area under curve values from receiver operating characteristic analysis of GWTG-HF score and its individual parameters. BP, blood pressure; COPD, chronic obstructive pulmonary disease.

Supplementary Table 2. Univariable Cox Regression Analysis

|  | **Mortality** | | **HHF or Mortality** | |
| --- | --- | --- | --- | --- |
| Parameter | Hazard ratio (95% CI) | p value | Hazard ratio (95% CI) | p value |
| GWTG-HF score (per 1 point increase) | 1.11 (1.07-1.17) | <0.0001 | 1.07 (1.04-1.11) | <0.0001 |
| Male gender | 1.76 (0.82-3.81) | 0.150 | 1.53 (0.88-2.67) | 0.131 |
| BMI (per 1 kg/m² increase) | 0.95 (0.87-1.03) | 0.229 | 1.01 (0.95-1.06) | 0.845 |
| History of smoking | 1.86 (0.84-4.11) | 0.123 | 1.26 (0.69-2.30) | 0.460 |
| Diabetes | 1.33 (0.59-2.98) | 0.492 | 0.93 (0.50-1.72) | 0.805 |
| Carotis stenosis | 1.68 (0.63-4.47) | 0.303 | 1.58 (0.76-3.26) | 0.219 |
| Peripheral artery disease | 1.76 (0.79-3.93) | 0.166 | 1.41 (0.79-2.50) | 0.247 |
| Prior stroke | 1.75 (0.66-4.65) | 0.261 | 0.96 (0.41-2.25) | 0.919 |
| Logistic EuroSCORE (per 1% increase) | 1.02 (1.001-1.04) | 0.038 | 1.02 (1-1.03) | 0.052 |
| NYHA (per 1 grade increase) | 1.30 (0.67-2.55) | 0.440 | 1.37 (0.84-2.25) | 0.206 |
| Atrial fibrillation | 0.64 (0.20-2.05) | 0.446 | 0.96 (0.37-2.52) | 0.937 |
| GFR (per 1 mL/min increase) | 0.96 (0.93-0.98) | 0.001 | 0.97 (0.95-0.99) | 0.001 |
| Nt-pro-BNP (per 1000 pg/mL increase) | 1.03 (1.003-1.06) | 0.032 | 1.02 (0.998-1.05) | 0.072 |
| Hemoglobin (per 1 g/dL increase) | 0.88 (0.74-1.05) | 0.167 | 0.95 (0.83-1.08) | 0.403 |
| Mitral regurgitation (per 1 grade increase) | 3.76 (1.96-7.20) | <0.0001 | 2.03 (1.29-3.21) | 0.002 |
| LVEF (per 1% increase) | 0.96 (0.93-0.99) | 0.003 | 0.96 (0.94-0.98) | 0.0004 |
| Systolic PAP (per 1 mmHg increase) | 0.99 (0.96-1.02) | 0.637 | 0.97 (0.95-0.997) | 0.030 |
| TAPSE (per 1 mm increase) | 0.91 (0.83-0.996) | 0.040 | 0.92 (0.86-0.98) | 0.007 |
| Tricuspid regurgitation (per 1 grade increase) | 1.53 (0.95-2.46) | 0.079 | 1.20 (0.84-1.72) | 0.309 |
| TTVR procedure | 0.55 (0.16-1.83) | 0.330 | 0.65 (0.29-1.46) | 0.300 |
| Procedural success | 0.77 (0.29-2.05) | 0.597 | 0.51 (0.27-0.96) | 0.038 |

BMI, body mass index; GFR, estimated glomerular filtration rate; LVEF, left ventricular ejection fraction; NYHA, New York Heart Association class; TAPSE, tricuspid annular plane systolic excursion; TTVR, transcatheter tricuspid valve repair.
